# Neurotransmitter patterns in patients with adolescent idiopathic scoliosis (AIS)

**DOI:** 10.1186/1748-7161-8-S2-O1

**Published:** 2013-09-18

**Authors:** Mark Morningstar

**Affiliations:** 1 Natural Wellness & Pain Relief Center, Richmond, MI, USA

## Background

Central nervous system function is dependent upon neurotransmitters to properly integrate midbrain and cortical function. Central nervous system dysfunction is thought to be a primary driver of AIS progression.

## Purpose

The goal of this study was to observe and analyze any patterns of imbalance in neurotransmitter status in patients with AIS.

## Methods

The charts of 31 AIS patients from the same multidisciplinary medical center, ages 11-18, were reviewed and compared to a sampling of 31 patients without a history of scoliosis. Testing consisted of a urinalysis panel, analyzing 12 common neurotransmitters.

## Results

Review of the completed urinalyses revealed a trend towards elevated histamine, elevated norepinephrine and decreased serotonin in the AIS patients as compared to non-scoliotics. Since these neurotransmitters are typically expressed in specific cortical areas, this pattern of imbalance may manifest as a functional hemisphericity, causing asymmetrical efferent responses to peripheral, afferent muscular inputs, and altering firing thresholds in their respective pathways.

## Conclusions and discussion

In the current sampling of patients with AIS, a trend toward specific neurotransmitter imbalances was observed. These imbalances may help shed light on the often-observed somatosensory dyscoordination seen in this patient population.
